# Experiences of the Medication Use Process by People with Intellectual Disabilities. What a Pharmacist Should Know!

**DOI:** 10.3390/pharmacy9010024

**Published:** 2021-01-21

**Authors:** Bernadette Flood, Martin C. Henman

**Affiliations:** 1 Daughters of Charity Disability Support Services Dublin, Dublin, Ireland; 2 School of Pharmacy and Pharmaceuticals Sciences, Trinity College Dublin, Dublin, Ireland; mhenman@tcd.ie

**Keywords:** intellectual disabilities, medication use, pharmacist, carer, quality, information transfer, medication risk, experience, health inequalities

## Abstract

There is a scarcity of information about the experience of people with intellectual disabilities in the medication use process. Six people with intellectual disabilities consented to be interviewed by a pharmacist to determine their knowledge and views of medication use. Data from semi-structured interviews were analysed using a grounded theory approach. Self-determination and risk to the quality of the medication use process were identified as theories. Literature review provided two explanations—vulnerabilities of people with intellectual disabilities in healthcare and pharmacists have a role to play in ensuring a quality medication use process for people with intellectual disabilities. People with intellectual disabilities may be ‘expert patients’ and can provide valuable insights into their experience of medication use. They may be ‘high risk’ patients but may not be recognized as such by pharmacists.

## 1. Introduction

The healthcare environment in which people with intellectual disabilities receive care and are prescribed medication is increasingly complex. The experience of all patients in healthcare is an accepted arm of quality. Patients say that they care about their experience of care as much as clinical effectiveness and safety. It has been suggested that some people with intellectual disabilities face a situation of ‘double jeopardy’ where they are at risk of receiving second rate services from both the disability and health service because they are seen to be the primary responsibility of neither [1]. Individuals with intellectual disabilities often depend on family members or other paid/unpaid, registered/unregistered decision makers for support with a wide range of healthcare decisions. The role of such ‘proxy’ decision makers is both important and challenging [2].

People with intellectual disabilities and their carers know the complexity of their needs and they alone know the real gaps in healthcare that can occur in services provided to them. They are the ‘experts’. Canadian “Consensus guidelines for primary health care of adults with developmental disabilities” [3] have highlighted the reality that adults with intellectual disabilities have a high risk of poor health and premature death owing in part to health disparities unique to adults with intellectual disabilities. It was hoped that the guidelines would increase primary care providers’ capacity to identify these disparities and address them through preventive and other health care interventions [4].

Intellectual disability means a significantly reduced ability to understand new or complex information and to learn and apply new skills (impaired intelligence) [5]. This results in a reduced ability to cope independently (impaired social functioning), and begins before adulthood, with a lasting effect on development. Seeking medical care, taking medications correctly and following prescribed treatments requires that people with intellectual disabilities and their carers understand how to access and apply health information. In healthcare locations and situations, people with intellectual disabilities and/or their carers are often faced with complex information and treatment decisions. Some of the specific tasks people with intellectual disabilities and/or their carers are required to carry out in the medication use process may include:

- evaluating information on medication for credibility and quality
- analyzing relative risks and benefits of any medication use
- calculating dosages of medications e.g., insulin, liquid antibiotics
- interpreting test results, e.g., blood sugar levels
- locating accessible, person centered and appropriate medication and health information e.g., ‘easy to read’ medication leaflets.

In IDS-TILDA (Intellectual Disability Supplement to the Irish Longitudinal Study of Ageing) https://www.idstilda.tcd.ie/, which is a large scale nationally representative study of people with intellectual disabilities aged 40 years and over in Ireland, more than half (56.5%) of participants reported they had never received ‘easy to read’ leaflets on keeping healthy and three quarters said they had never received ‘easy to read’ information leaflets on healthcare services [6]. A number of studies have indicated that people with limited health literacy have difficulty understanding written information, including medication dosage instructions and warning labels, discharge instructions, consent forms for treatment and basic health information about diseases, nutrition, prevention and health services [7]. Health literacy difficulties can affect the person with intellectual disabilities understanding of healthcare advice, how to take medication in a correct and safe manner, how to participate in self-care activities and how to make informed decisions about their healthcare. People with intellectual disabilities are unlikely to seek information about their medication, and if they are given information, they are likely to experience difficulties with both understanding and remembering what has been told to them about their medication. Even if they do possess some information, people with intellectual disabilities may experience difficulties in communicating about side-effects of their medication and they may have difficulties reporting adverse effects such as ‘blurred vision’ or feeling ‘dizzy’. It is also likely that carers may have only a limited knowledge of the medications which their clients are taking and of the possible side-effects [8]. They may be unaware that older people with intellectual disabilities and with mental health conditions can be exposed to high anticholinergic burden that is associated with daytime dozing and constipation [9].

Health literacy issues and ineffective communications place people with intellectual disabilities at greater risk than the general population of preventable adverse events involving medication use in healthcare. Oral language skills are important in health and social care for people with intellectual disabilities as all patients need to articulate their health concerns and describe their symptoms or side effects of medication accurately. Pertinent questions must be asked by the person with intellectual disabilities and/or their carer, and they need to understand spoken medical advice, directions for taking prescribed medications or treatment directions. If people with intellectual disabilities and/or their carers do not understand the implications of a medical diagnosis and the importance of prevention and treatment plans, or cannot access health care services because of communications problems, risk is created, and harm may occur.

The population with intellectual disabilities has been identified as being among ‘the most medicated groups in society’, with rates of prescriptions and polypharmacy exceeding those of the general population [10]. Paradoxically, a patient already exposed to polypharmacy may not receive other medicines due to challenges associated with communication and diagnostic overshadowing and fears of interactions with drugs already prescribed [11]. In a study of polypharmacy in older adults with intellectual disabilities in Ireland, almost half of the cohort reported pain, but only 2% used paracetamol-codeine combinations, and 1% opioids, while a third reported using paracetamol [12]. Pain and its appropriate treatment need to be examined in more detail in the population with intellectual disabilities. Pharmacists have a role to play in ensuring a quality medication use process for people with intellectual disabilities [13]. If pharmacists and other healthcare staff can engage people with intellectual disabilities and help them feel involved, they are more likely ‘to hear’ the advice they are given about medication and to act on it, so treatment is more effective and safer. Accessible and tailored information about medication must be discussed with people with intellectual disabilities in order to meet legal and best practice standards [14].

Too often, clinical services and programs are examined only on the basis of what matters most to the medical or other healthcare professionals; their concerns may relate to reduction of symptoms while service providers’ concerns may relate to costs. What matters most to people with intellectual disabilities and their carers may be functioning and quality of life. In Ireland and internationally, there is a dearth of research representing what is actually happening in the lives of people with intellectual disabilities in relation to medication use. Grounded theory methodology, which was used in this project, is particularly suited to looking at rarely explored phenomena where extant theory would not be appropriate. Grounded theory investigates their experience, the actualities in the real world and analyses the data with no preconceived ideas or hypothesis. This article describes a grounded theory approach to interviewing six Irish people with intellectual disabilities to establish their views and knowledge of medication use.

The aims of this qualitative study, using a Grounded Theory Approach, were to explore the experiences of participants with their medications and the medication use process and to discover how informed the participants with intellectual disabilities were about their medication and to identify key factors relating to their experiences and understanding of their medications and of the medication use process.

## 2. Materials and Methods

The researcher had worked for fourteen years as a pharmacist supporting people with intellectual disabilities and their carers in long term residential care and this provided a knowledge base that gave credibility to the analysis and interpretation of the data, allowing a deeper insight into the issues raised by the participants. The supervisor had 25 years’ experience in research, both quantitative and qualitative in pharmacy practice and in health services research. These learned skills facilitated good communication and ensured that the individual characteristics of the participants, the topic of the research project and any potential difficulties were considered and addressed in advance by the researcher and supervisor. The chief executive officer (CEO), medical director, and counsellor for a national support organisation for people with intellectual disabilities supported the project when approval had been received from the Faculty of Health Sciences Ethics Committee, Trinity College. The support organisation will not be named to protect the identity of the participants in the interviews.

The four ethical principles guiding this research project with people with intellectual disabilities are: (1) Non-maleficence—the research must not cause harm to the participants with intellectual disabilities and to people in general. (2) Beneficence—the research should make a positive contribution towards the welfare of people with intellectual disabilities. (3) Autonomy—research must respect and protect the rights and dignity of participants with intellectual disabilities. (4) Justice—the benefits and risks of research should be fairly distributed among people.

The original inclusion criteria for participation by people with intellectual disabilities in this project included: person has an intellectual disability, known to the national support organisation, taking medication, with ability to communicate verbally, aged over 40 years of age and with capacity to consent for themselves to participating in the project. The recruitment strategy and criteria were discussed by the researcher, the project supervisor and staff in the national support organization. There were many difficulties during the recruitment stage of this project as a number of support organisations acted as gate keepers, confirming some of the recognized barriers to recruiting people with intellectual disabilities to participate in research [15]. No person who consented to participate in this project was over 40 years of age. Six people with intellectual disabilities aged less than 40 years of age consented to participate. It was decided to continue due to the recognised difficulties of undertaking research in this population group and the importance to hearing people with intellectual disabilities describing in their own voice their experiences of the medication use process. It is recognized that there is a need to develop methods to enable the increased participation of people who have intellectual disabilities in some aspects of research [16].

This study involved 1–2-h long interviews, over 3 days, conducted face to face in two offices of an Irish national organization supporting people with intellectual disabilities and their carers. Due to ethical considerations, participants were recruited by the counsellor of the organization who distributed an accessible information leaflet describing the project, to people with intellectual disabilities, family members and staff in the organization. The researcher’s contact details were provided, and people were encouraged to contact the researcher with any queries they may have had. Contact was made by one family member. Six people with intellectual disabilities agreed to participate and signed accessible consent forms. The participants were informed on the Participant Information Sheet that the researcher was ‘interested in trying to find out what it is like to take tablets?’ and that she would like to speak to people who could answer that question.

The sole researcher clearly identified herself and established rapport with each person with intellectual disabilities prior to the individual interviews. The process was described for the participants and they were made aware that their rights, e.g., to anonymity and to stop the interview at any time of their choosing. The counsellor of the organization, who was familiar to each participant attended each interview as each participant expressed a desire that the counsellor be present. After each interview participants were encouraged to ask questions. The contact details of the researcher were given to each person in case they had further questions. No contact was made. The project researcher sent each participant a ‘Thank You’ card and a copy of their signed consent form. The counsellor confirmed the accuracy and the accessible nature of the language used in an information sheet of the project results that was posted by the researcher to each participant. Participants were invited to suggest any corrections/changes. No request for changes was made. Participants were sent a final ‘Thank You’ card. An abstract of the project was sent to the CEO, clinical director, and counsellor of the support organization.

To undertake research in the population with intellectual disabilities the researcher must establish rapport and trust if the participants are to tolerate her presence in their daily lives, to answer innumerable questions and to share intimate thoughts and beliefs [17]. To avoid verbal overloading, a semi-structured interview tool was used to guide the interviews. Sentences were short and dealt with one topic. Impairment of speech production is among the most commonly reported difficulties in children, adolescents and adults with intellectual disabilities [18]. The project did not receive approval, from the support organisation, for voice recording. People with intellectual disabilities may have trouble expressing their concerns, symptoms, thoughts or feelings as their cognitive impairment makes identifying, understanding and verbalising these difficult [19]. Some people may also have a coexisting physical condition that impacts on their speech. Short written notes were taken by the researcher during each interview. These were compiled, discussed with supervisor, and sent to the counsellor for verification that they were an accurate representation of what was discussed at each interview.

It is recognised that although this population is a difficult one to reach for research, attempts to do so should not be abandoned, because the potential health benefits for this underserved group far outweigh the recruitment barriers experienced [15]. The semi-structured individual face-to-face interviews used in this project were similar to structured interviews in that the topics or questions to be asked were planned in advance. However, instead of using closed questions, these semi-structured interviews were based on a mixture of open-ended questions (Appendix A).

Grounded theory aims to generate theory that is grounded in the data. It is ‘an inductive theory discovery methodology that allows the researcher to develop a theoretical account of the general features of the topic, while simultaneously grounding the account in empirical observation of data’ [20].

Several approaches to grounded theory exist. In this project the researcher ‘begins with an area of study and allows the theory to emerge from the data’ [21]. The Grounded Theory approach included the use of concurrent data collection and constant comparative analysis, theoretical sampling and memo-ing.

The qualitative design allowed for flexibility and for decision making to take place as the research process proceeded. Using this approach, the researcher aimed to enquire and to state how the six participants interpret reality of medication use and she was attentive to how theory emerged from the subjective experiences of the six participants. Data generation in this project showed that the participants provided additional direct, and indirect data next to the data gathered through semi-structured interviews, which was the main source. This additional data included lists of prescribed medications in three interviews, ‘brown bag’ medication and emotional impact of diagnosis and life situations.

Following the interviews, the data were read and reread by the single researcher to discover trends, themes and ideas. Independent assessment was provided by the research supervisor. Grounded theory highlights the importance of developing an understanding of human behaviour through a process of discovery and induction rather than from the more traditional quantitative research process of hypothesis testing and deduction. No extensive literature review was undertaken prior to this study because preconceptualising the problem, theoretical framework, or concepts that would follow from extensive literature review would have the potential to contaminate the emerging theory. The literature was treated like another source of data and was woven into the theory in the constant comparative process.

Memos were used as ‘the storehouse of ideas generated and documented through interacting with data’ [22] generated by the interviews. While interviews generated data, data sources also included documents and published literature. The relationship the researcher has with all her data, how it is generated and collected, will determine the value it contributes to the development of the final grounded theory [23]. The theoretical sensitivity of any researcher is deeply personal and reflects her level of insight into both herself and the area that she is researching. In this project memo-ing was an ongoing activity that built intellectual assets, fostered analytic momentum and informed the grounded theory findings.

All researchers are a sum of all they have experienced. It is expected that as a Grounded Theorist becomes immersed in their research data, their level of theoretical sensitivity to analytical possibilities will increase.

All participants were very personable and pleasant interviewees. They were interested and happy to participate in the process. Knowledge of their medication varied from moderate in the main to very little. Pseudonyms are used throughout; Pat, Alex, Keelan, Gabrielle, Jamie and Frances. Pat was identified as an index case in the analysis and his situation has been described in the literature [24]. All participants were able to consent to participate in this project and to verbalise their responses to the questions asked by the interviewer. It was not possible or necessary to identify their professionally assessed category of intellectual disability.

## 3. Results

Table 1 below sets out the medication data provided by the six participants.

The objective of this research was to generate new theory from data, as opposed to testing existing theory. The theories that emerged following the interview process included, (1) self-determination and, (2) risk to the quality of the medication use process. These theories drove the literature review and the extant literature is incorporated into the study as data.

### 3.1. Theory 1: Self-Determination

The Convention on the Rights of Persons with Disabilities (CRPD) [25] was signed by Ireland in 2007 and ratified in 2018. Self-determination is at the core of the CRPD along with the concepts of participation and inclusion. Self-determination refers to a characteristic of a person that leads them to make choices and decisions based on their own preferences and interests, to monitor and regulate their own actions and to be goal-oriented and self-directing. There is a growing literature base relating to self-determination and people with intellectual disabilities [26]. Research has shown that people with intellectual disabilities experience many fewer opportunities to make choices and express preferences across their daily lives. A review of the literature found that choice-making opportunity is a strong predictor of self-determination with evidence that the environments in which adults with intellectual disabilities live or work limit opportunities to make choices and restrict personal autonomy [27]. Promoting self-determination in health has been suggested as being a key strategy in reducing health inequalities [28].

The protection of patient self-determination entails the following elements [29]: (a) recognition of, and respect for, the right to decide what treatment to have or not to have; (b) provision of an enabling climate for the person with an intellectual disability to make self-determined choices (ensuring effective communication and building trust); and (c) having regard for the context (social, cultural, emotional, etc.) in which the person with an intellectual disability has to make his or her decision. Studies have shown that patients make treatment decisions based on their social experiences, emotions, relationships and values [30].

Alex reported that after starting trifluoperazine his ‘strength went down after taking tablets’ and he ‘found it hard to do things’. He appeared to have limited knowledge of individual tablets. However, he understood that trifluoperazine was used for a behaviour problem (screaming). He said his ‘parents know’ about his tablets. He also reported ‘no pain now’ while taking allopurinol. He reported that he ‘felt sick’ after taking some tablets. His doctor and his mother explain about tablets to him. He does not know his pharmacist and has not received any advice or ‘easy read’ material from the pharmacy.

The prescription and administration of medication is an area where people with intellectual disabilities experience a lack of control and disempowerment. His mother (a nurse), who requested to meet the researcher, expressed concern about further dose increases indicated by prescriber. She reported her experience of not being listened to by the prescriber. People with an intellectual disability are in the main compliant with medication prescribed for them despite their apparent lack of information and understanding. In one study despite participants with intellectual disabilities experiencing side effects, they were accepting of these effects. A ‘model of compliance’ was generated from that analysis [31]. Recently marginalisation of people with intellectual disabilities and their carers in decision making about psychotropic medication has been identified [32]. Marginalisation can be experienced during one of the three elements of being informed, being included and having influence.

Keelan reported that his parents were ‘wrecking my head’ and they are ‘driving me mental’ as they ‘have to know everything’. He reported having ‘lots’ of blood tests and that he experienced feeling like ‘a pin cushion’. He recalled being called a ’star patient’ and ‘the best patient in whole hospital’. He makes an appointment himself to see general practitioner (GP). He sees his GP alone who gives him the prescription. He ‘must show’ prescription to parents. He is accompanied by family members to the pharmacy where he reported that he does not get advice. He reported getting a ‘box of tablets in a bag’. If he does not want to take his tablets, he tells his parents who ’sometimes listen’.

Gabrielle’s experience is that she ‘deals with a lot’ of health matters herself. She was prescribed levothyroxine to help her ‘speed up’. She was dependent on her family to bring her to the pharmacy and medical appointments. She reported being ‘a good reader’ and reported that she had never been given ‘easy read’ material. Her experience was that the pharmacist will explain if there are ‘many things’. Gabrielle reported good things about tablets were ‘feel better’ and bad things were ‘thought of having to take tablets’ and ‘routine’. She reported that her tablets were her ‘own responsibility’ and that she has ‘spat out’ cod liver oil capsules after pretending to take them. If tablets make her ‘feel bad’ she will take them with a drink of water, lie down and ‘get a treat after’.

Jamie reported that she ‘knows all about medicines’. She appeared to be an expert patient with an expert carer who was a pharmacist. She reported being a diabetic who ’does not mind taking tablets or giving injections’. She is ‘used to it’. She reads leaflets to see about side effects. She would tell her mother if she did not want to take tablets or injections.

Pat reported that he hated being a diabetic and that he ‘hates the whole thing’ [24]. He reported using his insulin only because he was ‘not ready to die yet’. He reported storing his insulins in a drawer in his bedroom. He brought numerous insulin pens and glucagon injections to the interview with no indication of how long they had been removed from the fridge. Some of his medication was dispensed in a monitored dosage system (MDS) which had evidence of being used erratically. He had medications provided by two pharmacies. This may indicate his ‘self-determination’ to change pharmacy or a family member bringing a prescription to a pharmacy other that the pharmacy he regularly used. This use of multiple pharmacies adds risk to the medication use process. In this case Pat had the same medication (esomeprazole) provided by one pharmacy in original packs and the second pharmacy in an MDS. It has been suggested that prescribers and pharmacists should encourage patients to use a single pharmacy for safety purposes [33].

Four of the six people interviewed reported using non-prescription medicines including Vitamin B and Cod liver oil. It was unclear how and where they obtained these products. Supplement use has been reported to be almost twice as prevalent in the population with intellectual disabilities although substantially less diverse than the general population [34].

The experiences of all six participants in this project supported the central position of the family and/or individual family members in decision making about medications. Each person had a unique experience of self-determination relating to medication use and the medication use process. Limitation of choice and variation in the levels of control existed in the unique family environments. There is some evidence of a tension between promoting individual autonomy and the right to make decisions, and safeguarding, when people may make choices that are detrimental to their health possibly because they lack accessible information and/or capacity to weigh the future implications of their choices, and that uncertainty exists among carers about how to manage this tension [35].

### 3.2. Theory 2: Risk to the Quality of the Medication Use Process

To ensure patient safety it is important to identify risk. Patient safety consists of the identification, analysis, and management of patient-related risks and incidents, also called adverse events or medical errors, in order to make patient care safer and minimise harm to patients [36].

Compared to the general population with the relevant long-term condition, fewer people with intellectual disabilities and diabetes had a HbA1C record made in the previous 15 months, and fewer people with asthma and intellectual disabilities had an asthma review in the previous 15 months [37]. Medication use is the main therapeutic intervention in this population group [12] and the medication use process provides an opportunity for measuring the quality of care. Understanding the medication and supplement use of people aging with intellectual disabilities is critical to ensuring that health service providers in primary/ambulatory care can optimise use of these agents [34].

**Figure 1:**
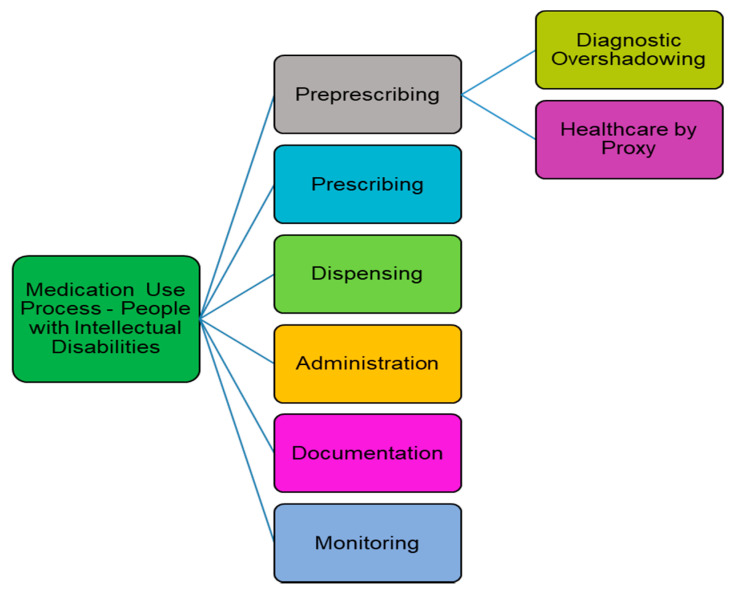
Medication Use Process—People with Intellectual Disabilities [41].

Several steps have been identified in the medication use process: prescribing, dispensing, administration, documentation and monitoring. The process as experienced by the population with intellectual disabilities includes a significant ‘pre prescribing’ step consisting of two important elements: recognition/assessment and diagnosis/cause identification, as shown in Figure 1. These are vital components of the medication use process experienced by this population with communication difficulties, in which ‘diagnostic overshadowing’ [38,39], and ‘healthcare by proxy’ [40], are significant factors. Both factors will introduce risk to the quality of the medication use process.

Direct care workers have a very critical role as advocates for health for the people with intellectual disabilities in their care [42] and their attitudes, skills and confidence will have an impact on the ‘preprescribing‘ stage and the quality of the other stages of the medication use process in this population group. Family members may also have this critical central role. Healthcare for people with intellectual disabilities contains an overshadowing bias when the presence of one impairment such as intellectual disabilities results in a reduced likelihood that another impairment will be identified. This ‘diagnostic overshadowing’ is a significant barrier to people with intellectual disabilities receiving quality healthcare. ‘Diagnostic overshadowing’ has been recognised by the National Patient Safety Agency (NPSA) in the UK as a patient safety priority for people with intellectual disabilities [43]. One barrier to healthcare in this population is the reliance of people with intellectual disabilities on health management ‘by proxy’ which involves numerous steps.

Medication management has emerged as the greatest problem area for social care providers according to Care Quality Commission Inspectors in the UK [44]. This is hardly surprising in the population with intellectual disabilities as the burden of therapy management, medication and supplement use and the potential risks in those ageing with intellectual disabilities differs substantially from those ageing without intellectual disabilities [9]. The interaction of organizational and human factors in the medication use process can result in risk for a person with intellectual disabilities in the medication use process. As these participants and their carers reported, people with an intellectual disability experience problems and their carers may be the first to notice any observable problem that arises from the medication use process [45]. Three risk areas associated with the medication use process, identified in this project include: (a) the transfer of medication information, (b) adherence to the medication regimen, and (c) side effects or ‘bad things’ about medicines.

#### 3.2.1. Medicines Information

Medication is a core part of the healthcare of many people with intellectual disabilities [11]. The right to consent to medical treatment, including medication use is a critical first step in promoting self-determination in health. It is therefore important to investigate the amount of knowledge people with intellectual disabilities have about their medication [14]. The NPSA in England has highlighted the lack of information about medication that people with intellectual disabilities have been prescribed as an area of concern [46] that will introduce risk to the medication use process. In this research project, Keelan and Alex both reported that they get a ‘bag of tablets’ in the pharmacy and do not receive any advice. Frances and Jamie however reported that they know their pharmacists who gives them written information. Gabrielle reported that the pharmacist will explain ‘if many things’ on prescription. Alex who understood that screaming could be described as a problem behaviour, was aware that trifluoperazine may be prescribed for a behaviour problem. He stated that his ‘parents know’ and that he has been ‘told’ to take medication by a parent. In psychiatric settings most people prescribed antipsychotics reported not feeling involved in treatment decisions and state that they take medication only because they are told to [47]. People in psychiatric settings reported that they had not been given written information about their treatment or warned about side effects, and stated that alternative non-pharmacological interventions had not been offered. Health professionals in primary care need education on the unique medical and pharmaceutical care needs of people with intellectual disabilities [3]. The relationship carers had with their pharmacist has been identified as central to the daily activities relating to ‘giving medicines’ to children with severe or profound intellectual disabilities [48]. A good relationship with a pharmacist can reduce risk in the medication use process.

#### 3.2.2. Adherence to Medication Regimen

Medication adherence may be defined as “the extent to which patients take medications as prescribed by their healthcare providers [49]. The participants in this project exhibited various levels of adherence with their prescribed medications. More complex medication regimens make self-management of medication more challenging and can negatively influence adherence [49]. Being non-adherent to prescribed medications may introduce risk to the medication use process.

Jamie appeared to be fully adherent because she understood her condition and the value of her medicines, insulin and monitoring regimen. Jamie stated that she ‘knows all about’ her own medicines and that she reads the leaflets to learn about side effects. Keelan reported ‘I don’t mind taking tablets’ and that he always takes his medicines and had no memory of ever forgetting to take them. Gabrielle always takes her medicines and feels better ‘now and again’ after taking them but this ‘depends how feeling’. However, she reported having pretended to take cod liver oil and that she ‘spat it out’ later without telling anyone as she has to ‘deal with a lot’ herself. Frances reported being ‘very sensitive’ and that she takes her tablets as they make her ‘feel better’. Frances noted that her medicines helped her to ‘feel more relaxed’ and they help her ‘to be positive’. Alex was adherent because he was ‘told to’ take his medicines. Pat, who had diabetes, and was ‘self-caring’ appeared to be the least adherent and does not like his medicines. If he does not want to take his tablets, he just does ‘not take them’ and has never spat out tablets or hidden them. However, Pat [24] brought a MDS for medication administration to the interview that indicated random and erratic consumption of his oral medication. He also brought numerous insulin pens of two types of insulin that he reported storing in a drawer in his bedroom. He reported having been administered glucagon injections for hypoglycaemia on numerous occasions which could indicate lack of control of his blood sugar levels.

Community pharmacists can have a major impact on patient adherence, as they are uniquely positioned to identify patients who may not be taking their medicines as prescribed, the reasons for this, and can intervene at the point of medication supply by providing education and counseling where appropriate [50]. Studies have shown that pharmacists can improve medication adherence rates, resulting in improved patient outcomes [51,52].

#### 3.2.3. Side Effects or ‘Bad Things’ about Medication

The terms “adverse effects” and “side effects” of medicines are frequently used interchangeably. An adverse effect is an undesirable harmful effect resulting from a medicine and a side effect is an effect secondary to the main or therapeutic effect of a medicine which may not necessarily be harmful and can be desirable in some circumstances. The term ‘bad things’ was used by the researcher and participants during the interviews to discuss ‘bad things’ about medicines and the medication use process.

The frequency of side-effects to medications may be greatly underestimated by doctors [53,54]. Adverse effects are a problem for at least half of those taking psychotropic medication and they may be a rational reason for the patient choosing to discontinue medication [55]. There is evidence that clinicians prescribing antidepressants only ask roughly one in five patients how well the drugs are working and only one in ten whether they are experiencing any side-effects [56,57].These questions are less likely to be asked of vulnerable groups [58] and people with intellectual disabilities. Individuals with intellectual disabilities and autism spectrum disorder prescribed psychotropic medications across multiple classes have experienced the most side effects with those prescribed only one class of psychotropic medication still displaying side effects [59]. Health guidelines for adults with intellectual disabilities suggest that continuing re-evaluation should ensure the least effective dose, the monitoring of side effects and the discontinuation of ineffective medications [3].

Alex reported his experience that his ‘strength went down’ and that it was ‘hard to do things’ after starting trifluoperazine [60] a phenothiazine antipsychotic. Documented side effects of this product include lassitude, muscular weakness and drowsiness. Alex’s mother reported an increase in weight, lethargy, problems sleeping and thirst. Dry mouth, appetite changes, weight gain, and difficulty falling asleep or staying asleep are recognised side effects. Neither Alex nor his mother were aware that trifluoperazine was an unlicensed preparation in Ireland at the time of prescribing and is still not indicated for this use at this time.

People with intellectual disabilities and their carers need information in a language they can understand. People with intellectual disabilities may not understand the term ‘side effects’ or not be able to share their experience of ‘side effects’. In this project the participants were able to identify ‘good’ and ‘bad’ things about medicines [41]. Keelan had never heard of ‘side effects’. Participant Gabrielle who was unaware of ‘side effects’ identified ‘feeling better’ as a ‘good’ thing about medicines.

Pharmacists should be aware that the vulnerabilities of people with intellectual disabilities in healthcare environments and processes [61] can result in risk to the medication use process. Pat had 13 Lantus^®^ insulin pens all removed from their original packaging. He also had three Glucagen Hypokits^®^ with no indication of when they had been removed from the fridge. He used a MDS erratically with no medications used in Week 1. The individual participants in this project were heterogenous with respect to abilities and disabilities. There will be different intervening conditions in each care environment and part of the medication use process that may introduce risk: family dynamics, carer status and confidence, education, qualifications, health literacy, resources, knowledge base (in intellectual disability health and social care) of the healthcare provider, e.g., pharmacist, GP.

### 3.3. Explanations

The initial theories of (1) self-determination and (2) risk to the quality of medication use process enriched by literature review generated two explanations.

Explanation 1: The person with an intellectual disability is vulnerable in the medication use process and self-determination during the medication use process by people with

- Is problematic for the person and their family, e.g., Keelan, Francis
- Is difficult to achieve but can be achieved with adequate support networks, e.g., Jamie
- May not ensure quality healthcare, e.g., Pat
- May lead to poor outcomes, e.g., Pat
- May increase risk of adverse events, e.g., Pat
- May increase vulnerability in the medication use process, e.g., Alex.

The recognition of side effects, i.e., ‘bad things’ and drug interactions experienced by a person with an intellectual disability will require a skilled and knowledgeable carer and/or professional. It is not known if the adverse effects reported by the participants in this project were previously reported to any other healthcare professional. Alex reported that his ‘strength went down’ and that he found it ‘hard to do things’ when taking trifluoperazine [41]. This could indicate akinesia which relates to loss of drive and energy and of having slowed down. Frances reported feeling ‘dizziness’ and having to get up slowly when taking aripiprazole.

Explanation 2: Pharmacists have a role in ensuring the quality of the medication use process for people with intellectual disabilities.

All six participants in this project lived with their families in the community and obtained their medication from community pharmacies. Pharmacists play a key role in managing patient and medication safety in the primary care setting and have the greatest opportunity compared with other healthcare professionals to improve patient and medication safety due to their frequent contact with the public [52].

Patients with intellectual disabilities are were at increased risk in the medication use process if they are not supported by a knowledgeable carer. Paid carers of people with intellectual disabilities have experienced being ‘out of the loop’ and being ‘ill equipped and dangerously exposed’ because they were responsible for administering and monitoring medication often without vital information concerning changes, doses, or effects [32]. The pivotal nature of supportive relationships for mothers who give medicines to their children with severe and profound intellectual disabilities, in particular GPs, hospitals and pharmacists has been recognized [25].

Limited provision of medicines information in accessible format was experienced by participants in this project. Communication of information to people with intellectual disabilities is context and person specific and each person with an intellectual disability will have a different receptive communication capacity. Therefore, communication strategies by prescribers and pharmacists and carers must be individualised.

In the expressive communication of individuals with intellectual disabilities there is a significant reliance on non-verbal communication, often to a greater degree than a typical adult in the general population would require. In particular the communication abilities and needs of individuals with intellectual disabilities and /or psychiatric illness are highly heterogeneous. Heslop, in a study of twenty one people with intellectual disabilities and their carers and prescribers living in four different regions of England, found that that few were fully informed about their treatment [62]. Many of their carers said that although they knew how to administer the medication, they knew little about why the person was taking it and what the implications might be. Heslop and her colleagues found the provision of information to people with intellectual disabilities and carers to be poor. They identified four key strategies to support people with intellectual disabilities in obtaining information about medication-spending more time providing and reiterating key information, providing accurate, up-to-date, accessible information about medications, providing training for carers in wider aspects of medication usage, and tailoring information to each person’s individual needs. Pharmacists incorporating these strategies into their interactions with people with intellectual disabilities and their carers may reduce risk in the medication use process.

## 4. Discussion

As far as the authors are aware this was one of the first attempts to undertake this type of research in Ireland. In this project the interviews uncovered ‘real life’ experience that six people with intellectual disabilities had of the medication use process. Self-determination and Risk to the Quality of the Medication Use Process emerged as theories capable of explaining the experiences of the participants. The combined participant data and literature showed that healthcare professionals, and pharmacists in particular could help people with intellectual disabilities more with their use of medicines.

The participants proved to be a valuable source of information relating to the quality of the process. Participant Jamie was a very knowledgeable participant who managed having diabetes and coeliac disease, with the support of her mother a pharmacist. She reported never having been admitted to hospital for complications of her diabetes and her situation contrasted strongly with the other participants. Gabrielle described how she spat her tablets out her cod liver oil capsules when she did not want to take them. Alex described how his ‘strength went down’ after taking antipsychotic medication. Pat [24] told the researcher that he stored his insulin in a drawer in his bedroom not in a fridge. The evidence from the MDS used by Pat indicated that he did not take his medication in a systematic manner and that he may not have understood how to use the MDS correctly. It is important for patients to understand the information about their medication to facilitate the management of health conditions [62] and store their medicines correctly.

As evidenced in this project, many people with intellectual disabilities have a self-determined wish to be active participants in their own healthcare. The levels of self-determination of the research participants varied with their unique family setting which can at times be problematic, e.g., Keelan—his family were ‘wrecking my head’ as they ‘have to know’ everything; and introduce risk to the medication use process e.g., Pat [24]. The situations of the six participants illustrated an interdependency in the lives of most people with intellectual disabilities as many people with intellectual disabilities live their lives relying on their family members and/or multiple professionals for support. As described in this project this interdependency may be obvious in accessing different aspects of healthcare such as GP and pharmacy visits where the participants rely on their families for transport to and from their doctors’ surgeries and their local pharmacies. Many of them also depended on their family member for support during healthcare encounters, for example when bringing the prescription to the pharmacy, e.g., Alex, Keelan, Gabrielle, and Jamie.

The grounded theory approach to the interview data provided risk to the quality of the medication use process as a second theory, with three specific risks areas of medicines information, adherence to medication regimen and ‘bad things’ about medicines. There is evidence from this research and other projects that services and clinicians may fail to adequately address safety issues and to understand the complex social networks on which people with intellectual disabilities rely. Good primary care identifies the particular health issues faced by adults with intellectual disabilities to improve their quality of life, to improve their access to health care, and to prevent suffering, morbidity, and premature death [3]. The pharmacists who supplied the insulin and MDS to Pat [24], may have been unaware that he was ‘self-caring’ and that Pat may not fully have understood how to use a MDS or the proper storage requirements for insulin. In community pharmacies time is often in short supply during healthcare interviews and interactions. However, providing a person with an intellectual disability such as Pat, with medicines, including insulin, alone is unlikely to be sufficient to achieve his treatment goals. Pharmacists must promote safety at the individual patient level [30].To address their medication-related needs, pharmacists need to accept greater responsibility for the outcomes of medicines use and should evolve their practices to provide their high-risk patients with intellectual disabilities, with individualized services. It is recognized that, healthcare providers (both primary and acute health care) may lack specialist training in this field [63]. During hospital stays and at discharge, it is critical pharmacists educate patients on how to take their medicines and to provide pertinent information on things such as drug-drug-interactions and drug-food-interactions in patient-friendly language [64]. Important factors for accessing health services for adults with intellectual disabilities are consistency of care and support, staff training, communication skills and time to communicate, and provision of accessible information [35]. Pharmacists’ interventions can range from the provision of written or oral information and counselling at the time of dispensing, to more complex interventions involving counselling, monitoring and support of patients’ self-management. Pharmacist counselling has been identified as a healthcare intervention directed to patients’ health-related needs that improve inter-professional and inter-institutional communication, by collaborating to integrate health services [65]. Pharmacists must develop a communication style that is patient centered and individualized in order to take greater account of the perspectives and experiences of people with intellectual disabilities and their carers while using their medication.

Four categories of complexity have been identified for primary care providers [66] that may be helpful for pharmacists interacting with people with intellectual disabilities. They include: medical (i.e., discordant conditions, chronic pain, medication intolerance, unexplained symptoms, and cognitive issues); socioeconomic (e.g., the unaffordability of medication, family stressors, and low levels of health literacy); mental health (e.g., depression or addictions resulting in poor medication adherence); and behavioural issues (e.g., anxiety about one’s symptoms). People with intellectual disabilities may be at increased risk of poor medication knowledge as they often experience additional challenges in health-related communication due to factors including lack of accessible information, poor communication skills of healthcare professionals and communication, and adaptive and cognitive difficulties associated with intellectual disabilities [63]. Keelan reported never having received any advice from his pharmacist. Gabrielle said that she knows her pharmacist, who explains if there will be delay, and takes the time to explain ‘if there are many things’. However, she reported never having received ’easy read’ or accessible information even though she is a ‘good reader’. Frances described getting a separate sheet of information with her medicines and also that it had ‘letters big enough’. Jamie reported that she receives ‘easy read’ leaflets from her doctor and that she reads the leaflets to learn.

The population with intellectual disabilities experiences health and healthcare inequalities [28], some of which could be alleviated by improving the basic competencies and level of training of all healthcare staff who care for people intellectual disabilities [67,68]. Patient safety issues in healthcare have also been raised by vulnerable people with intellectual disabilities themselves [69]. The NPSA has highlighted the lack of information about medication that people with intellectual disabilities have been prescribed as an area of concern [46]. The pharmacist is often ‘invisible’ in the care process for people with intellectual disabilities [70] and conversely, people with intellectual disabilities are also often invisible to pharmacists as they are a group of people that are ‘hard to hear’ and ‘hard to see’. People with intellectual disabilities who rely on their carer for support and to access health services and medicines may never visit the pharmacy. Pharmacists may therefore be unfamiliar with the communication and other challenges presented. One of the keys to managing this high-risk population and intervening to address health inequalities and address poor quality care processes involves making efforts to understand the situation of each person with an intellectual disability more fully. Clinicians such as pharmacists must try to understand the person’s social and economic situation which will significantly impact how a patient fares in the healthcare system, as well as when it comes to managing his or her health outside the system, in their living environment. Does the person manage their own healthcare, e.g., administration of medication or does their carer ‘take charge’? In this project, participant Gabrielle reported that her tablets were her ‘own responsibility’ while participant Alex reported that he was ‘told’ by his father to take his medicines. Interventions are needed to support carers to negotiate the tension between promoting self-determination and safeguarding against risk of medication harm when supporting the individuals with intellectual disabilities who may have ability to make autonomous informed decisions.

Accessible information provision has been identified as an area of concern by people with intellectual disabilities [46]. Four of the six participants in this project reported that they have never received accessible information from the doctor or the pharmacist. However even when information is provided to the person and/or their carer this does not guarantee that an individual patient/carer has understood and accepted the information they have received. Indeed, research is needed to clarify whether there are common strategies that are likely to improve the accessibility for a range of groups who need communicative support, or whether different groups have different requirements [71].

All participants in this study lived at home with family members. The potential of medication errors among the home health care population is greater than in other health care settings because of the unstructured environment and unique communication challenges in the home health care system [72]. Many patients in primary care have been found to take medications in ways that deviated from the prescribed medication regimen [73]. Participant Pat gave evidence of this by his non-compliance with the MDS in which his medications were supplied. Non-adherence to anti-epileptic medications has been identified as a potential medical risk for individuals with intellectual disabilities that is significantly impacted by the type of community living arrangement [74].The NPSA noted that more information is needed about medication incidents occurring in mental health and intellectual disability services to enable national learning to be derived. The NPSA found that some incidents related to confusion over what medicines patients had self-administered, possibly as a result of inadequate supervision or a lack of continued assessment of the patient’s suitability for self-administration [75]. There is evidence that frequent medication reviews and collaboration with other members of the health care team, especially pharmacists, will help to prevent adverse events associated with poor medication management [73].

Discrepancies exist between the number of adverse events reported in clinical settings versus the actual number of adverse events. In Ireland, it is recognised that there is a significant level of under reporting of clinical adverse events. However, medication adverse events were within the top three adverse events reported in 2012 [76] and represent 7.8% of all events reported. The Disability sector accounted for 8.6% of all medication related adverse events, 49.2% of all reports of violence/harassment and aggression and 11.8% of all falls reported. Understanding the underlying medication processes that lead to patient harm in the Disability sector and intervening to fix them will lead to improved safety for people with intellectual disabilities in all living environments.

Pharmacists and others should risk stratify their patients with intellectual disabilities to identify the right level of care and interventions for distinct subgroups of patients. This process will assign a risk status to patients, then using this information to target care and improve overall health outcomes. There is an ‘art’ in providing pharmaceutical care to vulnerable people with intellectual disabilities.

The Royal Pharmaceutical Society (RPS), in a Medicines Optimisation (MO) Briefing document focusing on people with intellectual disabilities intellectual disabilities [77], describes how pharmacists and other healthcare professionals can enable people with intellectual disabilities to improve their quality of life and outcomes from medicines use. The first principle of MO identified by the RPS in this context is ‘patient experience’ with the following steps:

- Understand the experience of the person and their carer; their priorities are not necessarily the same as members of their healthcare team.
- Ask if the person or their carer have any concerns or questions about their medicines and allow more time for the consultation.
- If taking medicines is a problem, explore routes or formulations with the person and/or carer to find a solution that works best for them.
- Be consistent in the supply of formulations and flavours as changes to these can cause uncertainty or distress.

These steps are reflected in the ‘real life’ experience of the medicines use process and the emotions it evoked as described by the six participants in this project. Pat, who ‘hated everything’ about his diabetes described taking his medication because he does ‘not want to die’. Alex’s mother had concerns about his prescribed psychotropic medications but did not feel listened to and was not recognized as an ‘expert carer’. Gabrielle has spat out cod liver oil capsules when she was unable to swallow them. She did not appear to have requested an alternate presentation that may have made the oil more palatable.

Patients with intellectual disabilities and their carers are major users of healthcare and social care. Their needs are complex. If scant attention is paid by pharmacists to their communication challenges, their literacy level, their manual dexterity, their care arrangements and their need for ‘reasonable adjustments [78]’ they are at risk of receiving suboptimal care and treatment. Patients with intellectual disabilities are unlikely to complain or raise issues with pharmacists and are ‘compliant’ in healthcare [31]. If their experience is unknown, they may have to accommodate inconvenience, inadequate care and treatment and inadequate information. This vulnerable population will require the expertise of pharmacists to ensure the quality of the medication use process.

### 4.1. Limitations and Strengths

This research project is the first attempt by a pharmacist in Ireland to understand the ‘real life’ experience of people with intellectual disabilities in the medication use process. How the data presented by the six participants was heard and interpreted will have been influenced by the researcher’s experience of working for fourteen years as a pharmacist in a residential care centre for people ageing with intellectual disabilities in the Dublin area. The participants who all lived at home with their families and were aged less than 40 years and were able to consent to participate. Their experience and knowledge of medication use will be very different to that of younger and older people with intellectual disabilities, those who are less or more able and those who live in supported or residential care.

Specific research process limitations arose from:

1. Difficulties accessing this population group, e.g., gate keeping by support organisations
2. Difficulties involved obtaining approval from university research ethics committee.
3. Voice recording not allowed by organization and university ethics committee.
4. No funding.

Strengths:

1. Pharmacist researcher had vast experience and interest in improving quality of medication use process in population with intellectual disabilities.
2. Supervisor very supportive, experienced in both quantitative and qualitative research, interested, and knowledgeable.

### 4.2. Research

1. Pharmacy must include perspectives of vulnerable populations and their carers/support personnel in future research. The practice of pharmacy must evolve to provide vulnerable patients with intellectual disabilities with individualised medicines-use services and processes. People with intellectual disabilities who take medicines, and their carers, know better than most what needs to change.
2. Analysis of medication safety incidents, associated with intellectual disability settings and people with intellectual disabilities, at national level could lead to recommendations for changes to clinical practice and processes.
3. Research is needed to clarify whether there are common strategies that are likely to improve the accessibility of relevant medicines information for people with intellectual disabilities who need communicative support.
4. The impact of disability on health and healthcare and the medication use process is rarely considered in healthcare policy and pharmacy research. Multi-centre studies to examine the value of providing individualized appropriate pharmaceutical care to the population with intellectual disabilities are required. Issues related to education and training of pharmacists in disability matters requires exploration. The role of specialist pharmacists to support generalist counterparts should be explored and their specific requirements for competencies and training.

## 5. Conclusions

Care of those people who are unable to care for themselves and compassion towards people who are vulnerable has been a basic doctrine of medicine since ancient times. The way a patient is treated as a person has for some time been seen as a cornerstone of quality.

Pharmacists are one of the most accessible healthcare professionals and can make a valuable contribution to the care of people with intellectual disabilities [13]. One of the most evidence-based avenues available to improve healthcare is to maximize the expertise and scope of pharmacists and minimize expansion barriers of the already existing and successful health care delivery model [79].

Pharmacists play a vital role in the areas of medication safety and management. Medication use is the main therapeutic intervention in the population with intellectual disabilities. Pharmacists are central to that process. People with intellectual disabilities require pharmacy and pharmacists to be ready and willingly to ensure people with intellectual disabilities experience a quality medication use process and the health service should support them in this process [80].

## Figures and Tables

**Table 1:** Medicines information provided by six participants with intellectual disabilities.

Participant Information						
Participant	Pat	Alex	Keelan	Gabrielle	Jamie	Francis
Lives at home	Yes	Yes	Yes	Yes	Yes	Yes
Age group (in years)	30–35	25–30	30–35	20–25	30–35	30–35
Gender	Male	Male	Male	Female	Female	Female
Home address	Outside Dublin	Outside Dublin	Outside Dublin	Outside Dublin	Dublin area	Dublin area
Diabetic	Yes	No	No	No	Yes	No
**Prescribed Meds**	**Yes**	**Yes**	**Yes**	**Yes**	**Yes**	**Yes**
Insulin Injections	Yes	No	No	No	Yes	No
Oral hypo-glycaemics	No	No	No	No	Metformin	No
Levothyroxine	Yes	No	No	Yes	No	No
Antidepressant	No	Escitalopram	No	No	No	No
Antipsychotic	No	Trifluoperazine	No	No	No	Aripiprazole
Gastrointestinal	Esomeprazole	No	No	No	No	No
Cardiovascular	Perindopril	No	Aspirin	No	Losartan	No
Gout	No	Allopurinol	Paracetamol	No	No	No
Hypnotic	No	Zopiclone 7.5	No	No	No	No
Anti-diarrhoeal	Loperamide	No	No	No	No	No
**Non-prescription Meds**	**Yes**	**No**	**Yes**	**Yes**	**No**	**Yes**
Analgesic	Solpadeine ~	No	No	No	No	Feminax ~
Vitamins/Minerals etc	No	No	Vit. B + C & Zinc	Vit B Cod Liver Oil	No	No
Antidiarrhoeal	Loperamide ~	No	No	No	No	No
Skin	No	No	No	Moisturisers	No	No
GIT	Domperidone	No	No	No	No	No

Not Their Real Names; ~ ‘as Required’/‘prn’, non-prescription preparations, available only from pharmacies in Ireland.

## Data Availability

Flood, B., The Specialist Pharmacist and Quality Indicators for Medication Use in People with Intellectual Disabilities and Behaviour Disorders (Unpublished), in Pharmacy and Pharmaceutical Sciences. 2016, Trinity College Dublin: Dublin. http://www.tara.tcd.ie/bitstream/handle/2262/83126/Flood,%20Bernadette_PhD%20Thesis%2029.08.16.pdf?isAllowed=y&sequence=1.

## Appendix A

People with an Intellectual Disability: Their views and knowledge of medication use.

Semi Structured Interview Questions.

Hello

Thank you for taking the time to meet me and to answer a few questions.

I am a pharmacist/chemist and I would like to get some information from you about your tablets/medicines.

1	What is your name?
2	What age are you?
3	Who do you live with?
4	Where do you live?
5	Do you take tablets/capsules/medicines?
6	Do you know their names?
7	What are the tablets/capsules/medicines supposed to do for you?
8	Who told you that you had to take tablets/capsules/medicines?
9	How many tablets/capsules/medicines do you take very day?
10	What time of the day do you take them?
11	Are there any rules/advice for taking the tablets/medicines? Time of day? Food? With water? Other?
12	Do you know your doctor?
13	Do you visit him/her often?
14	Tell me what happens when you visit your doctor. See Doctor See receptionist Blood pressure Weight Other
15	Do you get prescription/notes from the doctor?
16	Does someone go with you to visit your doctor?
17	Does the doctor give you information about your tablets/ capsules/medicines?
18	Do you know your chemist/pharmacist?
19	Do you go to the Chemist Shop/Pharmacy with the prescription?
20	Does someone go to the Chemist Shop/Pharmacy?
21	Tell me what happens when you go to the Chemist Shop/Pharmacy
22	Does your Chemist/Pharmacist give you information about your tablets/medicines? Yes No Sometimes Never
23	Can you open the tablet/medicine container?
24	Is the label easy to read?
25	Do you ever buy tablets/medicines in the Chemist Shop/Pharmacy without a prescription?
26	Tell me what happens when you wake up in the morning and have to take tablets/medicines
27	Do you like taking tablets/medicines?
28	Do you feel better after taking tablets/medicine? Yes No No change
29	Do you ever feel worse after taking tablets/medicines?
30	Do people/staff/family/friends explain to you about the tablets?
31	If you do not want to take the tablets who do you tell?
32	If the tablets/medicines make you feel bad/worse what do you do?
33	Who have you told about this?
34	Do you know what side effects of medicines are?
35	Did you ever have any side effects when you take medicines?
36	Have you ever spit out/hidden tablets/medicines?
37	Did tablets/medicines ever make you feel sick?
38	What happens if you do not want to take medicines/tablets?
39	Are tablets/medicines easy to swallow?
40	Do you ever visit a psychiatrist?
41	What does a psychiatrist Do?
42	Do you know what problem behaviour is? (Problem behaviour includes hitting other people, hurting yourself, being angry, screaming, breaking things)
43	Do you take tablets/medicines for problem behaviour? Yes Know Don’t know
44	What does that feel like?
45	Was anything else tried to help you with problem behaviour?
46	Can you tell good/bad things about taking tablets/medicines?
47	Can you remember what happened if you ever did not want to take tablets/medicines?
48	Do you ever have blood tests?
49	Who do you think know most about your tablets/medicines? Doctor Pharmacist Staff in organisation Nurse Friend You do Other
50	Who decides most about your tablets/medicines?

NB: Document used in practise allowed for answer to be recorded in writing during.
